# *T*_1_ Mapping for Myocardial Fibrosis by Cardiac Magnetic Resonance Relaxometry—A Comprehensive Technical Review

**DOI:** 10.3389/fcvm.2016.00049

**Published:** 2017-03-16

**Authors:** Christian R. Hamilton-Craig, Mark W. Strudwick, Graham J. Galloway

**Affiliations:** ^1^Centre for Advanced Imaging, University of Queensland, Brisbane, QLD, Australia; ^2^The Prince Charles Hospital, Brisbane, QLD, Australia; ^3^Medical Imaging and Radiation Science, Monash University, Clayton, VIC, Australia; ^4^Translational Research Institute, Brisbane, QLD, Australia

**Keywords:** *T*_1_ mapping, cardiac magnetic resonance, MRI, relaxometry, cardiovascular imaging

## Abstract

Cardiac magnetic resonance (CMR) imaging has been widely used to assess myocardial perfusion and scar and is the non-invasive gold standard for identification of focal myocardial fibrosis. However, the late gadolinium enhancement technique is limited in its accuracy for absolute quantification and assessment of diffuse myocardial fibrosis by technical and pathophysiological features. CMR relaxometry, incorporating *T*_1_ mapping, has emerged as an accurate, reproducible, highly sensitive, and quantitative technique for the assessment of diffuse myocardial fibrosis in a number of disease states. We comprehensively review the physics behind CMR relaxometry, the evidence base, and the clinical applications of this emerging technique.

Cardiac magnetic resonance (CMR) imaging has been used widely to assess myocardial perfusion and scar (1–5). It is the non-invasive gold standard for left and right ventricular quantitation, as well as the assessment and quantitation of focal myocardial fibrosis (after infarction or due to other causes of cellular injury). Myocardial necrosis causes high signal on late gadolinium enhancement (LGE) inversion recovery (IR) *T*_1_-weighted images with excellent signal-noise ratios, and this has become the reference standard for non-invasive scar imaging in cardiomyopathies of various causes (1–4). However, LGE is limited in its ability to assess and quantitate diffuse (non-focal) myocardial injury and fibrosis. LGE is affected by inconsistencies in acquisition parameters, such as choice inversion time (TI), and in post-processing when signal intensity (SI) thresholds may be arbitrarily applied to distinguish normal myocardium from fibrotic tissue (6, 7). Moreover, the critical issue with LGE is that SI is expressed on an arbitrary scale (*relative* SI compared to “nulled” normal myocardium). Imaging of myocardial fibrosis using *relative* differences between scar and normal myocardium tissue is therefore *qualitative*. Semi-quantitative analysis of LGE can be performed using signal thresholding applied to LGE images; however, there are differences in technique for infarct quantitation (8), and this is only relevant when regional scar/enhancement is present; it does not allow quantitation of diffuse interstitial fibrosis.

Thus, in non-ischemic cardiomyopathies, such as hypertension or diabetes, LGE CMR is unable to detect signal differential where the collagen deposition is diffuse and widespread throughout the myocardium (9).

## CMR Relaxometry

Cardiac magnetic resonance is an evolving technique, providing valuable and comprehensive data on the anatomy and functional integrity of both the heart and coronary blood vessels. Currently, CMR is performed at magnetic field strengths of 1.5 or 3 T. MR images generate by exploiting the magnetic property (called spin) of nuclei that have an odd atomic number or mass number (10). A proton generates a small magnetic field much like a bar magnet, because the proton has mass, a positive charge, and spins. This small magnetic field is referred to as its magnetic moment. The single proton of the hydrogen molecule gives it a significant magnetic moment and combined with its abundance in the human body, makes it an ideal marker for clinical MRI.

In the absence of an applied magnetic field, the magnetic moments of the hydrogen nuclei are oriented randomly; when placed in a high static magnetic field (*B*_0_) they will align either parallel or anti-parallel to the magnetic field. Spins that aligned parallel to *B*_0_ have a lower energy than those aligned anti-parallel, and therefore more align parallel creating a net magnetization (*M*_0_) of the sample in the direction of the magnetic field *B*_0_ (11).

### Larmor Equation

The interaction of a magnetic moment with *B*_0_ causes the magnetic moment to precess about the axis of the static magnetic field (*B*_0_), at a frequency specific to the strength of (*B*_0_)— the Larmor frequency. The Larmor frequency is defined as follows:

$$\nu =\frac{\gamma }{2\pi }B_{0},$$

where ν is the frequency, in megahertz, *B*_0_ is the strength of the magnetic field, γ is the gyromagnetic ratio for hydrogen, and γ/2π = 42.57 MHz/T (11).

When a radio frequency (RF) pulse at the Larmor frequency is applied to the nuclei within the magnetic field, nuclei begin to resonate and those in the lower energy state absorb energy. Depending on the RF pulse length, the precession of affected nuclei will be moved into the transverse magnetization plane (*xy* axis) and be in phase. With cessation of the RF field, the nuclei will realign to their original orientation parallel to *B*_0_—a process referred to as relaxation. During the relaxation process, the net relaxation induces an RF signal, at the characteristic frequency, which can be measured by a receiver coil. This signal is known as free induction decay (FID).

### MRI Relaxation Time

Three different properties of the interaction of the magnetic moments with *B*_0_ can be measured. These are the longitudinal time constant *T*_1_, or “spin-lattice” relaxation, and transverse time constant *T*_2_, or “spin–spin” relaxation and $T_{2}^{\;*}$, which is governed by a combination of the effect of spin–spin relaxation, and the homogeneity of the magnetic field. These constants are parameters that are used in MRI to distinguish between normal tissue types and pathological process. The SI of these times depends on the technical parameters that are used for image acquisition (12, 13) and the magnetic properties of a given tissue (14). At a given magnetic field strength, each tissue has a normal range for relaxation time. So, the variation of relaxation time from their normal value can be used to identify pathological process (e.g., edema and scar tissue).

*T*_1_ relaxation time refers to the tissue-specific time constant and is a measure of the time taken for protons to realign with the static field after perturbation by the RF pulse. This realignment with *B*_0_ is termed *T*_1_ longitudinal relaxation and the time in milliseconds. The above diagram *T*_1_ recovery curves demonstrate the fat has shorter *T*_1_ times than water molecules. This results from the fact that the fat nuclei lose their energy to lattice quickly, due to slow molecular motion, giving a relatively shorter *T*_1_ time. The quicker the system returns to the equilibrium state, such as occurs in fat, the greater the magnetization available to be excited by the next imaging pulse, producing more signals. Thus, fat appears bright in *T*_1_-weighted image (11).

**Figure 1:**
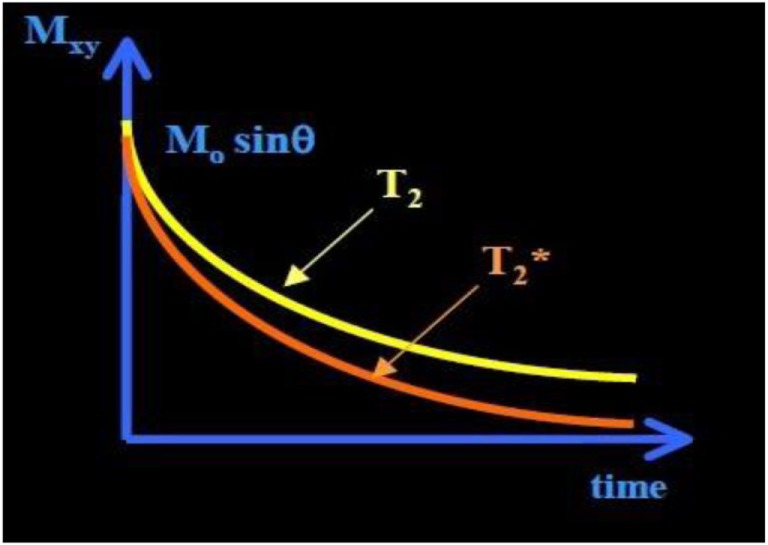
*T*_2_ and $T_{2}^{*}$ curve. The $T_{2}^{*}$ has shorter time than *T*_2_ time. $T_{2}^{*}$ exponential decay (≈30–100 ms, and shorter for higher *B*_0_).

*T*_2_ relaxation causes decay of signal arising from the dephasing of nuclear precession and consequent loss of net coherence (Figure 1) (15). *T*_2_ decay due to the magnetic interaction that occurs between protons, results in an exponential decay of the transverse magnetization vector, also governed by tissue structure. Unlike *T*_1_ relaxations, *T*_2_ does not involve a transfer of energy but only a change in phase. The water molecules have a long *T*_2_ decay and appear brighter in *T*_2_-weighted images due to the property that more rapidly moving molecules have a lower tendency to transfer their spin leading to a slower dephasing of the transverse magnetization.

A third decay parameter, $T_{2}^{\;*}$, describes the decay of transverse magnetization, due to spin–spin relaxation (*T*_2_), together with inhomogeneity in the static magnetic field (Δ*B*_0_), which occurs at tissue interfaces. This leads to a more rapid loss of phase coherence and the MR signal. These relaxation times are influenced by several factors, including field strength, blood iron content, blood volume, temperature, and blood oxygenation (15).

## Myocardial *T*_1_ Mapping in the Setting of Myocardial Fibrosis

The conventional *T*_1_ mapping method can be generated with an inversion recovery spin echo (IR-SE) sequence. Spin echo uses a 180°RF pulse to invert the spins within the selected slice, followed by a 90°RF pulse rotate the recovered magnetization at TI into the transverse plane, and a further 180° RF pulse to form the spin echo (12). Sampling of IR curve repeats multiple times with different TIs. The repetition time (TR) of the sequence must be long enough for recovery longitudinal magnetization before the next 180°inversion pulse. The SI of the final image is proportional to the relaxed fraction of the magnetization during the TI as follows:

$$\text{SI}={\mathrm{CM}}_{0}\text{ exp}\;\left(-\frac{\text{TE}}{T_{2}}\right)\;\left[1-2\text{ exp}\;\left(-\frac{\text{TI}}{T_{1}}\right)\right],$$

where the first exponential term relates to signal decay due to transverse relaxation (during TE), the second exponential refers to *T*_1_ relaxation (during TI). IR-SE is a gold standard to estimate *T*_1_ with a good accuracy. However, the drawback of IR-SE is that the TR of the sequence should be very long, i.e., TR should be at least five times longer *T*_1_ to allow full recovery of longitudinal magnetization. To reduce the acquisition time, inversion recovery turbo spin echo is currently used for routine clinical purposes (12).

On traditional *T*_1_-weighted images, the focal differences in *T*_1_ signal are measured qualitatively, assessed by visual inspection using relative units, and cannot be consistently compared between scans (16). For more precise measurement, an emerging technique for CMR *T*_1_ mapping has been applied to measure myocardial signal (in milliseconds) directly on a standardized scale within a single breath hold. This quantification of *T*_1_ mapping provides a benefit over the *T*_1_-weighted imaging of the myocardium by giving a quantitative measure of *T*_1_ time from multiple scans. A parametric map can be then reconstructed by calculating *T*_1_ values on a pixel-by-pixel basis (17), so, pixel intensities correspond to *T*_1_ values. Different cardiac MR acquisition sequences have been applied to create myocardial *T*_1_ maps, including Look–Locker (LL) and modified Look–Locker inversion recovery (MOLLI) (9, 16, 18–24).

### *T*_1_ Mapping Using the LL Technique

**Figure 2:**
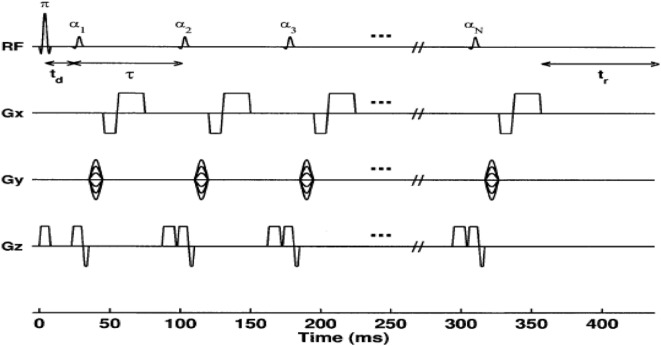
**Diagram of a conventional 2D Look–Locker pulse sequence**. The inversion-pulse/α-pulse train is repeated for every ky phase encode step. For *N* α-pulses, a series of *N* images are formed corresponding to times TI*_n_* = *t_d_* + (*n* − 1)τ(*n* + 1, 2, …, *N*) after the inversion pulse, where *t_d_* is the time between the inversion pulse and the first α-pulse (26).

The most common technique to measure spin-lattice *T*_1_ relaxation time values is the eponymously named “LL” sequence (also known as “TI scout”). It has been widely used to estimate the optimal TI for assessment of myocardial LGE (23, 25). It was originally proposed by Look and Locker in 1968 and developed more fully in 1970 (24). It consists of an initial inversion pulse, followed by a train of pulses with a constant, limited flip angle (7–15°). The inversion pulse prepares the longitudinal magnetization, which then recovers exponentially according to the *T*_1_ (24). The experiment is repeated until the *k*-space Cartesian map of data is filled, allowing an image to be reconstructed. By producing a train of absorption or dispersion signals (continuous wave magnetic resonance) or FIDs (pulsed magnetic resonance), it is possible to save time in spin-lattice relaxation measurements due to the fact that it is not necessary to wait for equilibrium magnetization before initiating the train (24) (see Figure 2). The total time required to acquire *T*_1_ would be significantly reduced compared to IR because the LL technique allows for multiple of *M_Z_* in a single measurement period (TM).

It was co-opted by Kaptein et al. in 1976 to quickly sample the recovery after a preparation pulse, during the recovery period or transient phase (27). This method was developed into the T one by multiple readout pulses (TOMROP) imaging sequence (28). In TOMROP, the multiple samples of a particular recovery after RF preparation each correspond to separate image. To acquire a complete data set for each image, the whole sequence must be repeated numerous times. Each repetition fills the next line of *k*-space for each image, and so on. Each image has a unique delay time. Early LL-based *T*_1_ techniques required the return to equilibrium of the spin system before the next application of an RF preparation pulse. Consequently, the acquisition time per slice of such implementations was long (28). Hinson and Sobol (29) used an LL method with no preparation pulse but the method suffered from poor accuracy, attributed to the slice profile. The late 1980s and early 1990s saw the LL method used for *T*_1_ measurements in a number of publications. Crawley and Henkelman (30) compared a number of one-shot and IR methods (LL, saturation recovery, IR, and stimulated echo) and concluded that the LL method was almost as efficient (in terms of dynamic range of the data and the proportion of the imaging time used to sample MR signals) as IR. Brix and colleagues used the TOMROP method with 32 gradient echoes to test for non-exponential behavior, found in fatty tissues (31), in a total acquisition time of 4 min. The LL single-shot IR method has been optimized and refined (32, 33) including improved RF preparation pulses (34, 35).

Echo-planar imaging (EPI) was incorporated into the IR LL-based method (36), by interleaving EPI readouts for eight different slices after an inversion pulse. The sequence was repeated, and the slice order was changed to achieve a range of TIs for each slice, with a total acquisition time of 30 s. LL with EPI was later applied *in vivo* in less than 3 s (37), using a modified blipped EPI technique (38), sacrificing and accuracy to some extent. An entire image was acquired at each point on a single recovery of longitudinal magnetization after a saturation pulse. The technique was optimized in 1998 (39) and has found applications in pharmacokinetic modeling (25).

The development of LL technique, which is available on Philips, GE, and Siemens platforms, is summarized in Table 1.

**Table 1:** **Summary of development of Look–Locker (LL) technique**.

**Reference**	**Summary of research findings**
Look and Locker (24)	Initial proposition of LL technique
Look and Locker (24)	Fully analyzed NMR pulse sequence to measure a spin-lattice *T*_1_ relaxation time
Kaptein et al. (27)	LL was co-opted to quickly sample the recovery after a preparation pulse during the recovery period
Gerumann (28)	T one by multiple readout pulses (TOMROP) was proposed in which the multiple samples of a particular recovery after radio frequency (RF) preparation each corresponds to separate image
Hinson and Sobol (29)	LL method was applied without preparation pulse
Crawley and Henkelman (30)	Compared [LL, saturation recovery, inversion recovery (IR), and stimulated echo] and concluded that LL was almost as efficient
Brix et al. (31)	TOMROP was used with 32 gradient echoes in a total acquisition time of 4 min
Kay and Henkelman (32)	LL single-shot IR method has been optimized and refined
Gowland and Leach (33)	LL single-shot IR method has been optimized and refined
Been et al. (34)	Improved RF preparation pulses
Gowland et al. (35)	Improved RF preparation pulses
Ordidge et al. (36)	Echo-planar imaging (EPI) was incorporated into the IR LL-based method
Gowland and Mansfield (37)	EPI was applied *in vivo* in less than 3 s
Freeman et al. (39)	An entire image was acquired at each point on a single recovery of longitudinal magnetization after a saturation pulse
Karlsson and Nordell (25)	EPI with LL method has found application in pharmacokinetic modeling in the head
Daniel et al. (40)	Modified Look–Locker inversion recovery (MOLLI) is proposed to overcome the limitations of the conventional LL approach for cardiac applications
Daniel et al. (22)	Studied the single breath-hold myocardial MR *T*_1_ mapping with MOLLI technique with high spatial resolution at 1.5 T MR reproducibility study
Daniel et al. (58)	Investigated optimization and validation of a fully integrated pulse sequence for (MOLLI) *T*_1_ mapping of the heart
Iies et al. (9)	Evaluation of diffuse myocardial fibrosis in heart failure with cardiac magnetic resonance contrast-enhanced *T*_1_ mapping

The LL sequence has been widely applied in CMR due to its fast acquisition with minimal breath-hold requirements. The LL sequence has been used to measure *T*_1_ values in patients with myocardial fibrosis (23). However, it suffers from significant limitations: low flip angle RF pulse exciting the magnetization and the two RR intervals in the LL sequence are not sufficient for the magnetization to return to equilibrium. This causes *underestimation* of true *T*_1_ values using LL. Furthermore, the LL *T*_1_ images with different TIs are acquired at different cardiac phases. Therefore, images are “cine” with cardiac motion effect, which requires tedious manually tracking of the myocardial borders for each phase, a labor-intensive and error-prone process which will is challenging in clinical practice. The drawing of regions of interest (ROI) in myocardial segments requires adjusting for cardiac motion, which result in including blood pool (partial volume averaging) and artificially increasing the measured *T*_1_ (40). *T*_1_ times between patients may vary due to differences in Gd kinetics (such as in renal impairment), or with different contrast agents; correction factors have been proposed using kinetic modeling for the LL technique (41).

To address these shortcomings, several myocardial *T*_1_ mapping sequences have been created, including MOLLI.

### *T*_1_ Mapping with MOLLI

Currently, the most evaluated sequence for myocardium *T*_1_ mapping is an MOLLI sequence (22, 42). The *T*_1_ mapping identifies a significant variation between normal and abnormal myocardium. It demonstrates that the myocardial fibrosis among different myocardial disorders includes ischemia (18), acute/chronic infraction (19), amyloidosis (20), diabetic (21), dilated and hypertrophic cardiomyopathy (17), and heart failure (9).

Modified Look–Locker inversion recovery is a CMR pulse sequence that is used for accurate *T*_1_ mapping of myocardium with high spatial resolution. A *T*_1_ map of the myocardium is a reconstructed image, where the *T*_1_ relaxation value is computed for every pixel of the corresponding myocardial voxel. Signal recovery from each myocardial voxel is acquired at different TIs following a single inversion pulse, all gated to the same cardiac phase, thereby enabling a pixel-based *T*_1_ quantification in the myocardium. MOLLI has introduced two variations to the standard LL sequence; selective data acquisition at a given time of the cardiac cycle over successive heartbeats, and merging of image sets from multiple LL experiments with varying TIs into one data set (22, 40). While selective data acquisition effectively decreases the number of images acquired in each LL experiment to one per heartbeat, the use of multiple LL experiments with different TIs increases the number of samples of the relaxation curve to a value that is sufficiently high for accurate *T*_1_ estimation.

**Figure 3:**
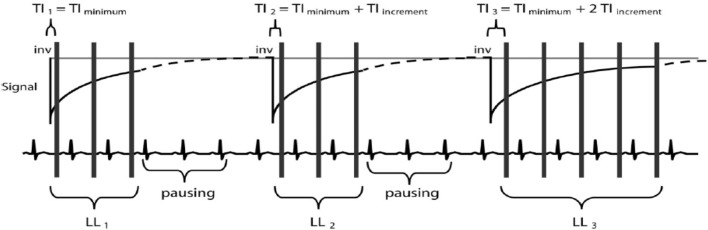
**Modified Look–Locker inversion recovery pulse sequence scheme**. There are three Look–Locker (LL) experiments, each prepared by a separate 180°inversion pulse (“inv”). The first is defined as TI_minimum_, and then TI of the second and third LL experiments is determined by TI_minimum_ − TI_increment_ and TI_minimum_ − 2TI_increment_. After inversion pulses, readout is in a non-segmented fashion with a single flip angle (α). A defined pause of a certain number of R–R intervals allows for signal recovery (40).

Modified Look–Locker inversion recovery is an ECG-gated pulse sequence scheme and uses three prepared LL experiments consecutively within one breath hold over 17 heartbeats to reconstruct 11 images with different TIs. Three successive ECG-triggered LL experiments (LL_1_, LL_2_, and LL_3_) are carried out with three, three, and five single-shot readouts, respectively, at end diastole of consecutive heartbeats to sample the recovery of longitudinal magnetization after the inversion pulse. MOLLI pulse sequence scheme is illustrated (Figure 3). *T*_1_ maps can be generated any time before or after contrast agent (e.g., gadolinium) administration (40).

**Figure 4:**
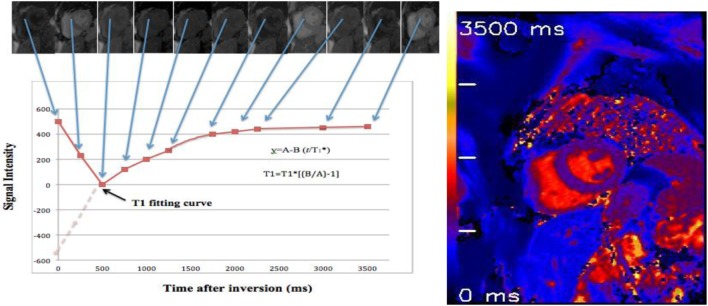
***T*_1_ map of a healthy volunteer: using 17 heartbeats to reconstruct 11 images with different inversion times (TIs) at end of diastole phase**. By merging these images into one data set, *T*_1_ values are computed for every pixel with three parameter curve fitting (39, 41). A reconstructed *T*_1_ map with parametric color scale is produced for these pixel values, and the segmental and global *T*_1_ times can be estimated.

Reconstruction of *T*_1_ maps from MOLLI source images is performed offline using purpose written customized software, or inline using vendor-specific processing, with ROI (septal or endo–epicardial) able to be analyzed in a pixel-wise quantitative fashion (Figure 4).

Image data are sorted by their effective TI, which are given by

$$t=\text{TI}+\left(n-1\right)\text{ RR},$$

where *N* = image number within the LL experiment and RR = heartbeat interval. Three-parameter non-linear curve fitting using a Levenberg–Marquardt algorithm is performed for corresponding pixel, which is given by

$$y=A-B\text{ exp}\;\left(-\frac{t}{T_{1}^{*}}\right),$$

where *y* denotes SI and $T_{1}^{\;*}$ corresponds to the apparent, modified *T*_1_ in an LL experiment.

Correction for readout-induced attenuation of the relaxation curve is attempted by using the three-curve fitting parameters $T_{1}^{\;*}$, *A*, and *B* for the calculation of *T*_1_. Then, *T*_1_ estimates as in conventional LL methods by

$$T_{1}=T_{1}^{\;*}\;\left(\frac{B}{A}-1\right),$$

The MOLLI sequence has been fully described, optimized, tested/re-tested, in phantoms and in large cohorts of healthy volunteers (22, 40) as well as being applied in cardiomyopathies (9, 17, 18, 20, 43). In addition, the *T*_1_ mapping with MOLLI has been validated against histopathology for assessment of myocardial fibrosis. It demonstrated that the pre-contrast “native *T*_1_” has a linear correlation with percentage of myocardial fibrosis as measured histologically on invasive myocardial biopsy. *T*_1_ times post-contrast administration (10–15 min) had an inverse linear relationship with collagen content in myocardial fibrosis subjects (9, 44, 45).

Nacif et al. (46) compared *n* = 168 myocardial *T*_1_ maps using LL and MOLLI at 1.5 T, showing that pre-contrast (native) *T*_1_ values had good agreement, but LL had wider limits of agreement, and post-contrast *T*_1_ maps also had good agreement but with LL giving higher values than MOLLI, hence they are not interchangeable (46).

*T*_1_ mapping can be generated for different segments of the myocardium (base, mid-cavity, and apex) within a single breath hold of about 15–20 s. However, the apex *T*_1_ values with MOLLI are slightly higher than basal and mid-cavity. The increasing in *T*_1_ values may be caused by partial volume effect and some degree of overestimation effect in apical level of left ventricle. These effects occur as a result of the ventricular wall being somewhat tilted toward the apex and no longer being aligned perpendicular to the short axis images (47–49).

Furthermore, *T*_1_ mapping with MOLLI has a greater reproducibility, accuracy, and an excellent overall inter- and intraobserver agreement over a wide range of TIs in pre- and post-contrast agent administration compared to the LL technique (22, 42). However, the *T*_1_ mapping with MOLLI sequence is sensitive to extremes of heart rate (bradycardia or tachycardia) (22) leading to slightly underestimation of *T*_1_ values. This may be corrected though “*heart rate correction*” by changing in the timing of the readouts with respect to the inversion pulses at different heart rates. This variation introduces various degrees of disturbance of the *T*_1_ relaxation curve. The heart rate affects the *T*_1_ value if the *T*_1_ value is higher than 750 ms or less than 200 ms with MOLLI (22, 42).

**Figure 5:**
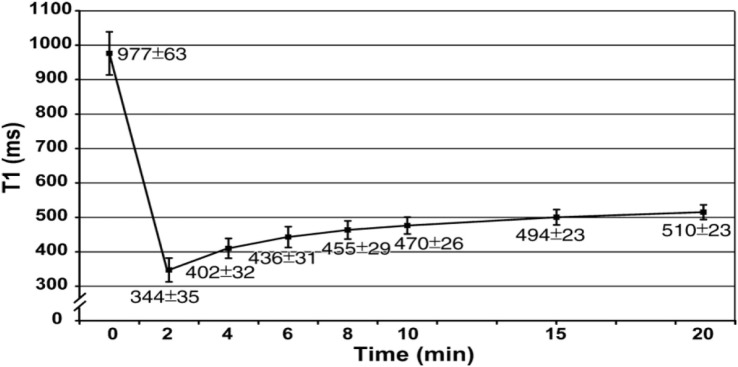
**Graph shows recovery of absolute *T*_1_ (mean ± SD in milliseconds) at 1.5 T in mid-cavity short axis slices at pre- and post-contrast (0–20 min) after administration of 0.15 mmol/kg of gadopentetate dimeglumine (22)**.

Moreover, MOLLI is limited by long breath hold about 15–20 s (17 heart beats to acquire the final *T*_1_ maps). This may be difficult for elderly and pulmonary compromised patients and generates respiratory and motion artifacts (50). However, modern inline processing provides registration tools to reduce motion artifacts before the computation of final *T*_1_ maps (motion corrected or “MoCo MOLLI”). This will minimize the sensitivity of *T*_1_ mapping to motion artifacts and heart rate. Also, various acquisition sequences with short breath hold, such as shortened modified Look–Locker inversion recovery, have been validated and recently applied for cardiomyopathies (51, 52). At 1.5 T, the pre- and post-contrast (10 min) *T*_1_ times of normal myocardium are 980 ± 53 and 470 ± 26 ms, respectively (Figure 5) (22). Pre-contrast *T*_1_ values of myocardial fibrosis (infarction scar) are significantly longer than those of normal myocardium (1,060 ± 61 vs. 987 ± 34 ms) (43). The longer pre-contrast *T*_1_ values in myocardial fibrosis patients have been reported in different cardiomyopathies. Infarction, myocarditis, and interstitial diffuse fibrosis, all have high pre-contrast *T*_1_ values when compared with normal myocardial *T*_1_ times (17, 20, 43). However, longer *T*_1_ values may also be noticed in different pathologically important processes, such as edema (53). Previous phantom, animal, and human tissue based studies lend insight into the effects on *T*_1_ signal by showing that *T*_1_ increases with increased water content and amount of extracellular fibrillar macromolecules (14, 54). Messroghli et al. concluded that increased myocardial *T*_1_ mapping corresponds to the areas of the human myocardial infarction, which showed on LGE (43). However, the links between the exact molecular mechanism in healthy tissue and myocardial fibrosis and corresponding pre-contrast *T*_1_ are less well understood. On the hand, the decrease in post-contrast *T*_1_ values in diffuse myocardial fibrosis has been previously related to increased extracellular space. It has been well described in acute, chronic ischemic, diffuse myocardial fibrosis, and inflammatory myocardial injury. The post-contrast *T*_1_ times (10 min) were significantly shorter in chronic infarct scar compared with normal myocardium at 0.15 mmol/kg (390 ± 20 vs. 483 ± 23 ms, respectively) (43).

Also, *T*_1_ mapping with high magnetic field (3 T) has been reported in a few studies of interstitial myocardial fibrosis. It was similar to 1.5 T, the pre-contrast *T*_1_ was longer, and post-contrast *T*_1_ was shorter in myocardial fibrosis patients compared to normal myocardium. Puntmann et al. (55) reported higher pre-contrast *T*_1_ values for hypertrophic and non-ischemic-dilated cardiomyopathies at 3 T compared to controls (hypertrophic 1.254 ± 43 ms and non-ischemic-dilated cardiomyopathy 1.239 ± 57 ms vs. healthy 1.070 ± 55 ms). Also, the post-contrast *T*_1_ values (10 min) at 3 T were shorter in hypertrophic and dilated cardiomyopathies compared to healthy (hypertrophic: 307 ± 47 ms, dilated cardiomyopathies: 296 ± 43 ms vs. controls: 402 ± 58 ms) (55).

There are studies published for normal and diffuse myocardial fibrosis of myocardium *T*_1_ values, as described comprehensively in Tables 2 and 3.

**Table 2:** Healthy clinical studies using *T*_1_ and $T_{2}^{*}$.

Reference	Sample size	*T*_1_/$T_{2}^{*}$ mapping sequence	Result of *T*_1_ or $T_{2}^{*}$ mapping (ms)
Wacker et al. (56)	5	srTFL, segmented $T_{2}^{*}$ gradient echo pulse	*T*_1_ = 1,219 ± 72 ms
			$T_{2}^{*}$ = 35 ± 3 ms
Sebastian et al. (57)	12	LL	*T*_1_ = 1,033 ± 126 ms
			$T_{2}^{*}$ = NA
Messroghli et al. (22)	15	MOLLI	*T*_1_ = 980 ± 53 ms
			$T_{2}^{*}$ = NA
Messroghli et al. (58)	20	MOLLI	*T*_1_ = 939 ± 63 ms
			$T_{2}^{*}$ = NA
Sparrow et al. (59)	15	MOLLI	*T*_1_ = 980 ± 53 ms
			$T_{2}^{*}$ = NA
Iles et al. (9)	20	VAST	*T*_1_ = 975 ± 62 ms
			$T_{2}^{*}$ = NA
Li et al. (60)	13	2 echo times GRE	*T*_1_ = NA
			$T_{2}^{*}$ = 33 ± 6.5 ms
Reeder et al. (61)	5	Multi-echo GRE	*T*_1_ = NA
			$T_{2}^{*}$ = 38 ± 6 ms
Anderson et al. (62)	15	Multi-echo GRE	*T*_1_ = NA
			$T_{2}^{*}$ = 5,216 ms
Positano et al. (63)	15	Multi-echo GRE	*T*_1_ = NA
			$T_{2}^{*}$ = 38 9.2 ms in endocardial sectors and 33.1 ± 8.4 ms in epicardial sectors
Messroghli et al. (64)	20	Multi-echo GRE	*T*_1_ = NA
			$T_{2}^{*}$ = 27.9 3.4 ms in anteroseptal and 23.1 ± 5.2 ms in inferolateral
Piechnik et al. (51)	342	shMOLLI	*T*_1_ = 962 25 ms
			$T_{2}^{*}$ = NA
			Heart rate only physiologic factors effect on myocardial *T*_1_ values

*NA, not applicable; srTFL, saturation recovery turboFLASH; LL, Lock–Locker; MOLLI, modified Lock–Locker inversion recovery sequence; VAST, inversion recovery gradient echo sequence with variable sampling of the *k*-space in time; GRE, gradient pulse sequence; shMOLLI, short modified Look–Locker sequence*.

**Table 3:** **Clinical studies using *T*_1_ mapping for myocardial diffuse fibrosis in clinical patients**.

Reference	Cardiac disease category	Patient sample size	*T*_1_ mapping method	Summary of findings
Thuny et al. (66)	Systemic sclerosis	37	Modified Look–Locker inversion recovery (MOLLI)	LV diastolic dysfunction had a shorter 15 min post-contrast *T*_1_ time (ms) than those with a normal diastolic function (431 ± 7 vs. 464 ± 8, *p* = 0.01)

Thibault et al. (67)	Type II diabetic patient	24	MOLLI	Mean myocardial *T*_1_ relaxation time was significantly shorter in diabetic patients than in volunteers both at 5 (312 ± 5 vs. 361 ± 6 ms, respectively, *p* < 0.001) and 15 min (405 ± 6 vs. 456 ± 5 ms, respectively, *p* < 0.001) after gadolinium injection

Ellims et al. (68)	Hypertrophy cardiomyopathy	51	VAST	Post-contrast myocardial *T*_1_ time was significantly shorter in patients with HCM compared to controls, consistent with diffuse myocardial fibrosis (498 ± 80 vs. 561 ± 47 ms, *p* < 0.001)

Kammerlander et al. (69)	Patients with NH_2_-terminal portion of the precursor of brain natriuretic peptide (NT-proBNP)	37	N/A	In patients with NT-proBNP levels >400 pg/ml mean *T*_1_ was significantly shorter than in patients with NT-proBNP <400 pg/ml (374.6 ± 51.1 vs. 404.6 ± 34.4 ms, *p* = 0.042) and controls (509.4 ± 46.5 ms, *p* < 0.001)

Sibley et al. (70)	Non-ischemic cardiomyopathy	73	Look–Locker (LL)	47 patients had a focal myocardial scar and 26 without scar tissue. The midwall circumferential strain (Ecc) was reduced (–13.0 ± 5.4%), and mean *T*_1_ time was 478 ± 70 ms in patients with no scar tissue

Jellis et al. (21)	Type II diabetic patients	67	VAST	Subjects have a shorter post-contrast *T*_1_ = 434 ± 20 ms. Post-contrast *T*_1_ was associated with echocardiography diastolic dysfunction (Em *r* = 0.28, *p* = 0.020; E/Em *r* = −0.24, *p* = 0.049)

Messroghli et al. (42)	Acute myocardial infarction	8	Inversion recovery (IR)-prepared fast gradient echo sequence	*T*_1_ pre-contrast value of the infarcted myocardium was significantly prolonged compared with non-infarcted normal myocardium (+18 ± 7%). *T*_1_ 10-min post-contrast value of the infarct was significantly reduced compared with normal myocardium (−27 ± 4%)

Messroghli et al. (43)	Acute and chronic myocardial infarction	24	MOLLI	In chronic MI, the pre-contrast *T*_1_ relaxation time of hyper-enhanced areas was higher than *T*_1_ of remote areas (1,060 ± 61 vs. 987 ± 34 ms, *p* < 0.0001). In acute MI, the pre-contrast *T*_1_ value of hyper-enhanced areas was higher than remote areas (1,197 ± 76 vs. 1,011 ± 66). The hyper-enhanced in acute is higher than chronic infarction

Sebastian et al. (57)	Acute and chronic myocardial infarction	10	LL	Mean *T*_1_ values of the normal myocardium post-contrast was 536 ± 66 ms; chronically infracted pre-contrast and post-contrast was 1,000 ± 67 and 408 ± 43 ms, respectively

Sparrow et al. (59)	Myocardial fibrosis in chronic aortic regurgitation	8	MOLLI	There is a significant difference in segmental averaged *T*_1_ relaxation between in abnormal wall motion vs. normal control segments in 10, 15, and 20 min after administration, Gd: 510 vs. 476, 532 vs. 501, and 560 vs. 516 ms, respectively

Iles et al. (9)	Chronic heart failure	25	VAST	Post-contrast myocardial *T*_1_ times were shorter in heart failure subjects than controls (383 ± 17 vs. 564 ± 23 ms) controls even when excluding areas of regional fibrosis. *T*_1_ 15-min post-contrast values correlated significantly with collagen volume fraction on myocardial biopsies (*R* = –0.7)

Maceira et al. (71)	Cardiac amyloidosis	22	Segmented IR sequence	Subendocardial *T*_1_ in amyloid patients was shorter than in controls (at 4 min: 427 ± 73 vs. 579 ± 75 ms; *p* < 0.01)

The myocardium is made of densely packed myocytes, contributing to approximately 90% of myocardial mass. Signal from pre-contrast imaging reflects the majority of signal from the myocytes themselves. Conversely, in post-contrast imaging, the majority of Gd-based contrast signal arises from the interstitium (as Gd is an extracellular contrast agent and does not, therefore, reside within myocytes unless they are damaged); thus, post-contrast *T*_1_ mainly reflects interstitial or replacement fibrosis (see Box 1, below), hence the ability to calculate extracellular volume (ECV) from the difference between these two parameters.

Box 1 **Source of *T*_1_ signal.**

*Pre*-contrast “Native” *T*_1_ = predominant signal from *myocytes* (replacement fibrosis, or intracellular accumulation, e.g., Fabry disease). *Post*-contrast *T*_1_ = predominant signal from *interstitial* space (interstitial fibrosis).

### Extracellular Volume

*T*_1_ mapping data can be used to calculate ECV fraction, using the pre- and post-contrast *T*_1_ times and hematocrit. Details of this technique and its uses are beyond the scope of this review; SCMR guidelines exist to guide application of *T*_1_ mapping and ECV quantitation (65).

### Limitations of *T*_1_ Mapping

Challenges remain with myocardial relaxometry for *T*_1_ mapping. These include technical challenges such as variations of *T*_1_ times at different field strength and across different vendors, and the rapidity in growth of pulse sequences being released as product and as works-in-progress, calling into question both the inherent accuracy and the level agreement between these techniques. Furthermore, the variations in *T*_1_ relaxometry values with different contrast doses and image timing require further investigation, to establish the test–retest and inter-site reproducibility of this technique. Next, the challenges to application of *T*_1_ mapping to clinical practice include establishment of robust normal ranges in large cohorts across multiple ethnic groups, and the observation that *T*_1_ mapping appears to be a highly sensitive technique, with the ability to discriminate healthy normal myocardium and identify very early changes in substrate. However, this technique lacks specificity; a wide variety of conditions prolongs native *T*_1_ and/or shortens post-contrast myocardial *T*_1_. Therefore, further clinical data are required in order to establish the use of these parameters in relation to disease (e.g., early detection of target organ damage in systemic conditions such as hypertension or diabetes), to inform treatment decisions, and their ability to predict or alter clinical outcomes.

## Conclusion

Myocardial *T*_1_ mapping using quantitative relaxometry is an emerging and important tool in the assessment of global myocardial fibrosis. It is a highly sensitive marker of disease but is not specific, with changes in myocardial *T*_1_ occurring in many different conditions. Nevertheless, the high sensitivity and excellent reproducibility of the technique offers a tool for the early detection of myocardial damage, over-and-above techniques such as the CMR LGE technique and other modalities such as speckle tracking echocardiography, pulse wave velocity, and tissue tagging. Native *T*_1_ mapping is proving to be a robust indicator of early myocardial disease in many conditions, and normal ranges and guidelines for post-processing have been published by the Society of Cardiovascular Magnetic Resonance (65). Myocardial *T*_1_ mapping is a rapidly evolving technique, now with longitudinal prognostic data emerging, and normal ranges established at 1.5 and 3.0 T in healthy humans and in aging. Further questions remain as to the standardization of pulse sequences across field strengths and between vendors, the affect of contrast type, dose and timing, the post-processing software, and the interpretation of *T*_1_ mapping results to inform clinical practice.

## Author Contributions

CH-C, MS, and GG: main contributions to the conception or design of the work; the interpretation of data for the work; drafted the work and revised it critically for important intellectual content; final approval of the version to be published; and agreement to be accountable for all aspects of the work in ensuring that questions related to the accuracy or integrity of any part of the work are appropriately investigated and resolved.

## Conflict of Interest Statement

The authors declare that the research was conducted in the absence of any commercial or financial relationships that could be construed as a potential conflict of interest.
